# Nonspecific back pain in adolescents, its associated physical and psychological factors and urban–rural differences: a cross-sectional analytical study

**DOI:** 10.3389/fpubh.2026.1780994

**Published:** 2026-03-13

**Authors:** Gauri A. Oka, Ashish S. Ranade, Ashwini S. Bodas, Aruna B. Deshpande

**Affiliations:** 1 Central Research and Publication Unit, Bharati Vidyapeeth (Deemed to be University) Medical College, Pune, India; 2 Blooming Buds Center for Pediatric Orthopaedics, Deenanath Mangeshkar Hospital and Research Centre, Pune, India; 3 Maharashtra Medical Foundation’s Joshi Hospital, Pune, India

**Keywords:** adolescents, nonspecific back pain, physical, prevalence, psychosocial, screen time, urban–rural

## Abstract

**Background:**

The school bag weight is a frequently implicated cause of back pain (BP) in children. Researchers from high-income countries have highlighted the contributions of physical and psychosocial factors, along with ergonomics and screen time exposure. However, almost all Indian studies on BP in children have focused on the school bag weight only, without clinical examination. Our objective was to estimate the prevalence of nonspecific BP and identify the associated physical and psychological factors in urban and rural adolescents.

**Methods:**

We conducted a cross-sectional study on students from 5th, 7th, and 9th grades from urban and rural schools across five districts of an Indian state. We documented students’ anthropometry, the school bag weights, physical factors, psychological factors (using the Strengths and Difficulties Questionnaire), and screen time exposure. A pediatric orthopaedic surgeon clinically examined students reporting BP to rule out specific causes.

**Results:**

We included 1,225 children [653 (53.3%) boys]. The prevalence of BP was 472/1225 (38.5%). Clinical examination of those with BP confirmed 441/472 (93.5%) students had nonspecific BP. More urban students reported BP (43.4% Vs. 33.2%, *p* < 0.001). A greater proportion of students with BP had some/high need in the emotional (OR 1.8, 95% CI 1.3 to 2.4), conduct (OR 1.7, 95% CI 1.3 to 2.2), hyperactivity (OR 1.8, 95% CI 1.3 to 2.6), and peer problem (OR 1.2, 95% CI 1.0 to 1.6) domains. Multivariate regression showed that some/high need in the SDQ hyperactivity domain [OR 2.05, 95% CI 1.1 to 3.6], mobile use of ≥ 60 min [OR 1.67, 95% CI 1.1 to 2.4], using computers [OR 1.4, 95% CI 1.0 to 1.9], presence of a family member with BP [OR 2.91, 95% CI 2.1 to 3.9] and history of back injury [OR 7.46, 95 CI 4.2 to 13.0] were significant risk factors of BP. Rural adolescents attributed BP to domestic and farm work, while urban adolescents attributed it to heavy school bags.

**Conclusion:**

Prevalence of nonspecific BP in adolescents is substantial. Apart from school bag weight, several physical and psychological factors and urban–rural differences exist. There is a clear need for tailor-made interventions to address these factors.

## Background

Back pain poses a significant public health challenge in today’s world, not only in adults but also in children. The lifetime prevalence of back pain in children is variable, ranging from 5 to 89% based on large studies as well as a systematic overview describing nonspecific or idiopathic back pain in adolescents from Europe and Australia (1–3). Back pain may limit a child’s physical activity and lead to absence from school, may increase the chances of back pain in adult life, and is a risk factor for chronic pain (4). Back pain has also been shown to affect the quality of life of a child adversely (5–7). Back pain in children continues to attract the attention of researchers, with the international consortium of spine societies worldwide (SPINE20 global advocacy group) hailing “children and adolescent spine” as one of the areas of immediate concern (8). Apart from a spectrum of various medical conditions, the current evidence points towards a combination of other risk factors, such as biological (female sex, overweight-obesity), lifestyle-related (being sedentary), and psychosocial factors (1, 9, 10). In addition, it has been found that a history of parental back pain is associated with back pain in children (10, 11). A majority of the studies have described physical factors contributing to back pain. Although important, only some studies have considered the role of psychological factors such as emotional, conduct and hyperactivity problems along with physical factors such as excessive screen time exposure (1, 11, 12). There are very few studies on back pain in children from India. They are mainly on urban children and focus on the weight of the school bag (13). They lack psychological evaluation and clinical examination (13, 14). Less than 30% of children with back pain seek medical care (5), highlighting the need to clinically examine children with back pain in the community setting itself. We conducted the present study with the objectives to estimate the prevalence of nonspecific back pain in adolescents, to determine the associated physical and psychological factors and urban–rural differences.

## Methods

**Figure 1:**
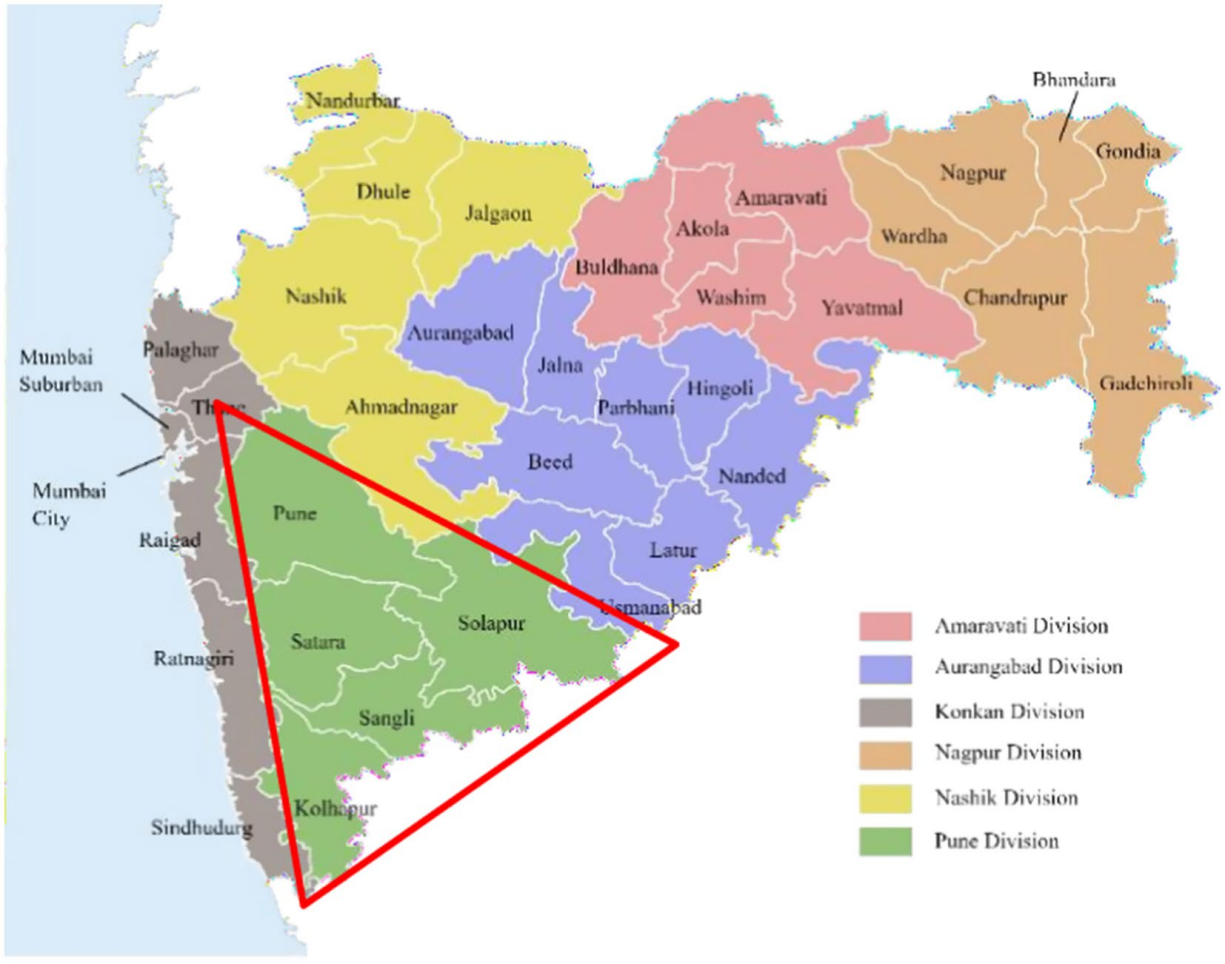
Study area: five districts of Pune Division (outlined within the red triangle), Maharashtra State, India.

Our study was approved by the Institutional Ethics Committee. The data collection took place between August 2022 and September 2023. The state of Maharashtra in Western India is divided into six divisions. This cross-sectional study was conducted in schools from five districts (Pune, Satara, Sangli, Kolhapur, and Solapur) of the Pune Division (Figure 1).

**Figure 2:**
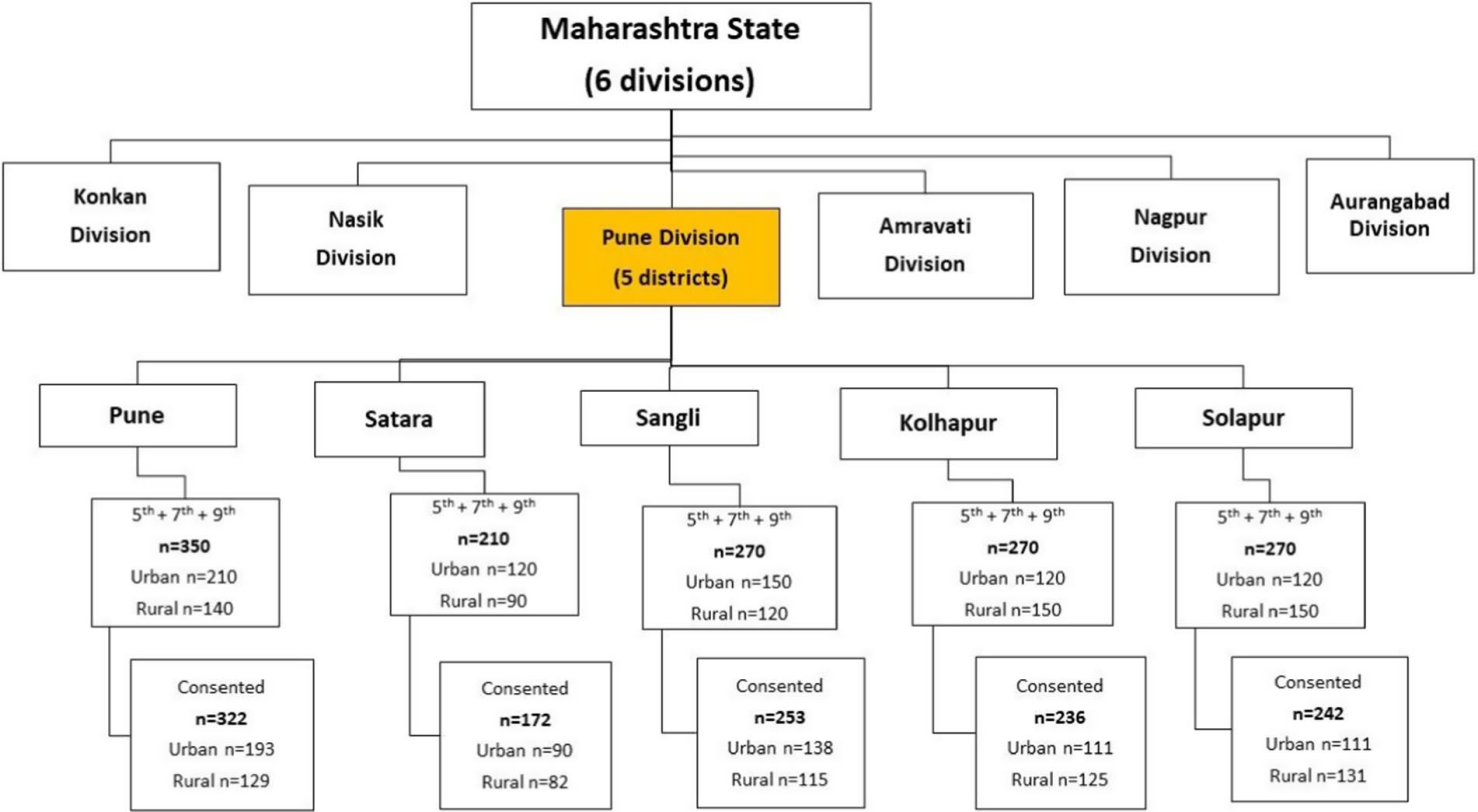
Sampling structure.

Schools were approached using non-probability sampling. One rural and one urban school from each district (10 schools in all) were included after acquiring permissions from the respective school Principals. Students from fifth, seventh, and ninth grades (chosen as representatives of the adolescent age group) were included. As reported in an earlier study (11), the prevalence of back pain was 53.9%. Allowing for a provision of a “no-consent” response rate of 20%, the minimum required sample size was calculated to be 459. Considering an average, typical class strength of 40 students, we decided to include the first division in alphabetical order that had at least 40 students (e.g., if there were 35 students in division A and 40 in divisions B and C each, division B was included). At the time of the first visit, child assent forms, parental consent forms, and printed information sheets detailing the study procedures were distributed in the classes and sent home with the students. After allowing 8–10 days for responses, the date for data collection was fixed. On the day of data collection, students were included in the study if they produced both, the signed assent and parental informed consent forms. The final sample was included as shown in Figure 2.

The investigators explained all the study-related procedures to the students, including height and weight measurement, school bag weight measurement, and guidelines for answering the questionnaires. A proforma was used to document the age, sex, school grade, school bag weight, and students’ heights and weights. The height was measured to the nearest 0·1 cm using a portable stadiometer. A calibrated digital scale was used to measure body weight and school bag weight to the nearest 0·5 kg. Body weight was measured with the participant barefoot and after removing heavy clothing items. A month’s recall of back pain was documented. The proforma included a diagram for marking the location of back pain and a visual analog scale (VAS) to note the pain severity. Details of back pain, such as frequency, duration of an episode, treatment sought, absence from school, aggravating factors, and perceived reasons were recorded. ‘Upper back pain’ was defined as pain up to and above the level of the spine of the scapula. ‘Middle back pain’ was defined as the area between spine of scapula and a horizontal line passing through the lowermost border of the last rib, and ‘lower back pain’ was defined as the region below the horizontal line passing through the lowermost border of the last rib. These operational definitions for back pain location were used as described in an earlier study (11). Psychological factors were recorded using the self-administered English Strengths and Difficulties Questionnaire (SDQ) for ages 4 to 17 years. It is a 25-item questionnaire and provides assessment on five scales, namely, prosocial behavior (strength), and emotional problems, conduct problems, hyperactivity, and peer problems (difficulties) (15). Each item can be marked as “not true,” “somewhat true,” or “certainly true.” The cut-offs of scores to define “low,” “some” or “high” need of support are as follows: Conduct and peer problems are scored as 0–3 (low need) and 4–10 (some to high need), hyperactivity and emotional problems are scored 0–5 (low need) and 6–10 (some to high need). The prosocial behavior is scored in a reverse manner with 6–10 (low need) and 5 and 0–4 as some and high need of support, respectively. The English self-reported SDQ was translated into Marathi by a professional translation agency. This was scrutinized and approved by a linguist with fluency in both Marathi and English, after which it was back-translated into English by the same agency. The back-translated version was finally critiqued and approved by the linguist. This version was used for rural schools.

Each student was required to document a week’s self-reported average daily screen time spent on each visual display unit, namely, mobile phones, television (TV), and computers or laptops. The total daily screen time per student was calculated by adding the durations across all devices.

A pediatric orthopedic surgeon conducted a comprehensive review of the history and a detailed on-site clinical examination of students with back pain to rule out specific causes. Physical examination included inspection for local swelling and external deformity. Inspection in a standing posture was done to assess the spine alignment. Palpation was carried out to assess paraspinal muscle spasm, tenderness, and step-off in the spinous processes. The range of motion of the spine was checked, including flexion and extension, and whether associated with pain and stiffness. A forward bending test was done to evaluate for scoliosis. A scoliometer examination was performed while the student was stooping forward. A neurological examination was performed that included motor and sensory function evaluation. A straight leg raise test was done to look for nerve root tension signs. In addition, the flexion-abduction-external rotation test was done to evaluate the sacroiliac joint. Based on history and physical examination, imaging was suggested whenever a specific cause was suspected. Nonspecific back pain was determined based on the absence of any of the above signs. Whenever the SDQ scores warranted attention, the respective class teacher and the school counselor, wherever available, were notified.

## Data analysis

Data were analyzed using SPSS version 28 for Windows package (IBM SPSS Statistics for Windows, Version 28.0. Armonk, NY: IBM Corp). Continuous data were analyzed as means and standard deviations (SD) and categorical data as proportions (%). Associations were determined using the chi-square test (or Fischer’s exact test, as applicable). Multiple logistic regression was employed to determine the significant predictors of back pain based on the significant factors identified in bivariate analysis. Odds ratios and 95% confidence intervals (CI) were obtained for each predictor. *p*-values of < 0·05 were considered significant.

## Results

Signed consent and assent forms were returned by 1,225 out of 1,370 students (response rate 89.4%) across 10 schools. There were 643 (52.5%) urban students, and 653 (53.3%) were boys. The mean age of students in the study was 12.4 ± 1.6 years (range 9.2 to 16.6 years), the mean weight was 39.5 ± 12.7 kg (range 16.0 to 96.6 kg), the mean height was 147.6 ± 11.6 cm (range 109.0 to 181.6 cm) and the mean body mass index (BMI) was 17.8 ± 3.8 kg/m^2^ (range 10.8 to 35.9 kg/m^2^). The mean school bag weight was 4.8 ± 1.5 kg (range 0.5 to 12.5 kg), and the mean school bag weight as a percentage of body weight was 13.1 ± 5.0% (range 1.4 to 41.2%). The weight of the school bag was found to be greater than 10% of the student’s weight in 895/1225 (73.1%) students. The proportion of students reporting a month’s recall of back pain was 472/1225 (38.5%), of which 239 (50.6%) were boys. The characteristics of back pain are shown in Table 1. All 472 students reported back pain on 1 to 3 occasions in the preceding month, with 396/472 (83.9%) reporting mild to moderate intensity of pain.

**Table 1:** Characteristics of back pain (*n* = 472).

Characteristic	*n* (%)
Location	
Upper back	178 (37.7)
Middle back	33 (7.0)
Low back	130 (27.5)
Combination of 2 or more areas	131 (27.8)
VAS scores (intensity)	
1–3 (mild pain)	136 (28.8)
4–6 (moderate pain)	260 (55.1)
7–10 (severe pain)	76 (16.1)
Frequency	
1–3 times in the last month	472 (100.0)
Duration of an episode	
Up to 2 days	432 (91.5)
>2 days	40 (8.5)

VAS, visual analog scale.

A small proportion of students reported taking medicines for their back pain (41/472, 3.3%), and 56/472 (4.6%) reported remaining absent from school because of back pain. There was no difference in the mean ages of students with and without back pain (12.4 ± 1.6 Vs. 12.3 ± 1.6 years, respectively, *p* = 0.775). Significantly more urban students (279/643) reported back pain as compared to their rural counterparts (193/582); (43.4% Vs. 33.2% respectively, OR 1.5, 95% CI 1.2 to 1.9; *p* < 0.001), as did students with a history of back injury (81.0% Vs. 33.7% respectively, OR 8.4, 95% CI 5.3 to 13.3; *p* < 0.001), when there was a family member with back pain (57.2% Vs. 27.6% respectively, OR 3.5, 95% CI 2.7 to 4.4; *p* < 0.001), those carrying school bags weighing more than 10% of their body weight (40.9% Vs. 32.1% respectively, OR 1.4, 95% CI 1.1 to 1.9; *p* = 0.005), and those who cycled or were driven to school as compared to those who walked (40.4% Vs. 34.6% respectively, OR 1.2, 95% CI 1.0 to 1.6; *p* = 0.048). Significantly more students who participated once a week in school sports reported back pain as against two or more days per week (45.5% Vs. 37.1% respectively, OR 1.4, 95% CI 1.05 to 1.9; *p* = 0.021). We found no association between back pain and the sex of the student, the student’s BMI, whether the bag was carried on the back or not, and whether the bag was carried on one or both shoulders. This was true even when comparing urban and rural students. The distribution of students with and without back pain according to the extent of psychological needs is shown in Table 2.

**Table 2:** Difference in the proportion of adolescents with back pain with “some/high” and “low” need of support across the SDQ domains.

SDQ domain (*n*)	Adolescents with back pain with some/High need *n* (%)	Adolescents with back pain with low need *n* (%)	OR (95% CI)
Emotional problem (1201)	124/247 (50.2)	339/954 (35.5)	1.8 (1.3 to 2.4)
Conduct problem (1194)	169/352 (48.0)	293/842 (34.8)	1.7 (1.3 to 2.2)
Hyperactivity (1197)	70/134 (52.2)	393/1063 (37.0)	1.8 (1.3 to 2.6)
Peer problem (1193)	184/433 (42.5)	277/760 (36.4)	1.2 (1.0 to 1.6)
Prosocial behavior (1200)	67/157 (42.7)	396/1043 (38.0)	1.2 (0.8 to 1.7)

SDQ, Strength and Difficulties Questionnaire.

**Figure 3:**
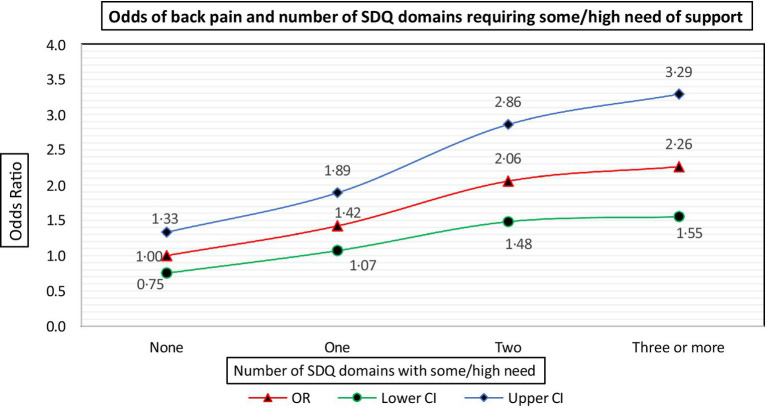
Odds of back pain and number of SDQ domains requiring some/high need of support.

A significantly greater proportion of students with back pain had some/high need of support in the emotional, conduct, hyperactivity, and peer problem domains compared with those having low need in these domains. Also, the odds of back pain were significant and increased with a student having some/high need in an increasing number of SDQ domains (Figure 3).

**Figure 4:**
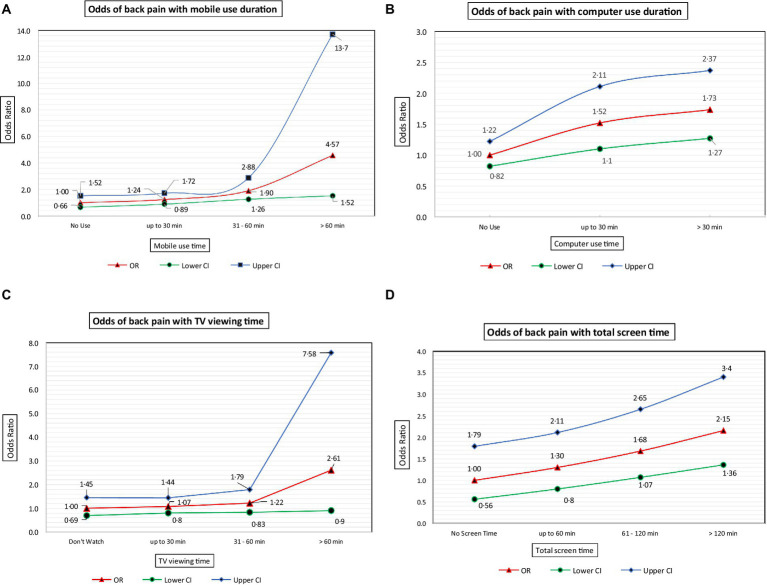
**(A)** Odds of back pain with increasing duration of mobile phone usage. **(B)** Odds of back pain with increasing duration of computer usage. **(C)** Odds of back pain with increasing duration of TV viewing time. **(D)** Odds of back pain with increasing duration of total screen time.

Figures 4A–C depict the odds of back pain with increasing durations of exposure to various visual display units, namely mobile phones, computers, and television (TV).

The odds of back pain were significant and increased beyond 30 min each of mobile phone (Figure 4A) and computer usage (Figure 4B). Although not statistically significant, the odds of back pain increased with increasing TV viewing time (Figure 4C). As shown in Figure 4D, the odds of back pain were significant and increased beyond 60 min of total screen exposure time.

We compared the characteristics of urban and rural children with back pain (Table 3).

**Table 3:** Differences in the characteristics of urban (*n* = 279) and rural adolescents (*n* = 193) with back pain.

Characteristic	Urban % or mean (SD)	Rural % or mean (SD)	OR (95% CI)	*p*-value
Age	12.6 (1.6)	12.2 (1.5)	–	0.010
Males	42.3	48.2	–	0.376
BMI	18.8 (4.3)	16.8 (3.4)	–	<0.001
Bag weight	5.6 (1.5)	4.5 (1.3)	–	<0.001
% of students carrying >10% of their body weight	76.3	79.3	–	0.453
Bag weight as % of body weight	14.1 (5.6)	13.2 (4.2)	–	0.074
Back injury	25.1	16.6	1.7 (1.05 to 2.7)	0.027
Family member with back pain	56.3	52.3	–	0.398
Taken medicines for back pain	6.8	11.4	–	0.082
Absence from school due to back pain	9.3	15.6	1.4 (1.04 to 1.8)	0.038
VAS score	4.4 (2.2)	5.1 (2.2)	–	<0.001
Total screen time	124.1 (80.0)	143.9 (108.2)	–	0.023
SDQ emotional problem score	3.7 (2)	4·6 (2.2)	–	<0.001
SDQ conduct problem score	3.0 (1.7)	3.2 (1.7)	–	0.221
SDQ hyperactivity problem score	3.6 (1.8)	3.4 (1.8)	–	0.292
SDQ peer problem score	3.2 (1.6)	3.0 (1.6)	–	0.279
SDQ prosocial score	7.5 (2.0)	8.1 (1.8)	–	<0.001

We found several significant differences. Rural children had a lower mean BMI (kg/m^2^) (16.8 ± 3.4 Vs. 18.8 ± 4.3; *p* < 0.001). They carried significantly lighter bags (kg) (4.5 ± 1.3 Vs. 5.6 ± 1.5; *p* < 0.001). Back injury was more prevalent in urban children (25.1% Vs. 16.6%; OR 1.7, 95% CI 1.05 to 2.7, *p* = 0.027). The mean total screen time of rural children was longer, they had worse mean emotional problem scores and better prosocial scores than their urban counterparts with back pain. In response to the question about perceived reasons for their back pain (Table 4), 81/193 (41.9%) rural and 152/279 (54.3%) urban children felt it was the “heavy school bag.” This difference was significant (*p* = 0.008). A significantly larger proportion of rural children, compared to urban [26 (13.5%) Vs. 6 (2.1%); *p* < 0.0001], perceived that their back pain was due to their engagement in domestic chores like washing utensils, fetching water, sweeping floors, or toiling on the farm.

**Table 4:** Differences in urban (*n* = 279) and rural (*n* = 193) adolescents’ perceived reasons for back pain.

Perceived reason for back pain	Urban *n* (%)	Rural *n* (%)	*p*-value
Heavy school bag	152 (54.5)	81 (41.9)	0.008
Faulty posture	60 (21.5)	35 (18.1)	0.365
Sports/exercise	32 (11.5)	8 (4.1)	0.004
Domestic work/farm work/ difficulty in getting to school	6 (2.1)	26 (13.5)	<0.0001
Miscellaneous (injuries, menses, being sedentary, excessive mobile phone use, etc.)	24 (8.6)	11 (5.7)	0.237

Clinical evaluation of 472 students with back pain identified 31 (6.5%) students with a suspected specific cause and were advised appropriate radiographs. One student followed up with the radiograph, which was normal. In this group of 31, 10 (32.2%) students had a history of acute back injury, and 25 (80.6%) reported activity limitations and muscle spasm. Moderate to severe pain was reported by 26 (83.9%), and 9 (29%) had night pain and had taken medicines. In three students, the scoliometer reading was abnormal (>5^0^), suggestive of scoliosis.

**Figure 5:**
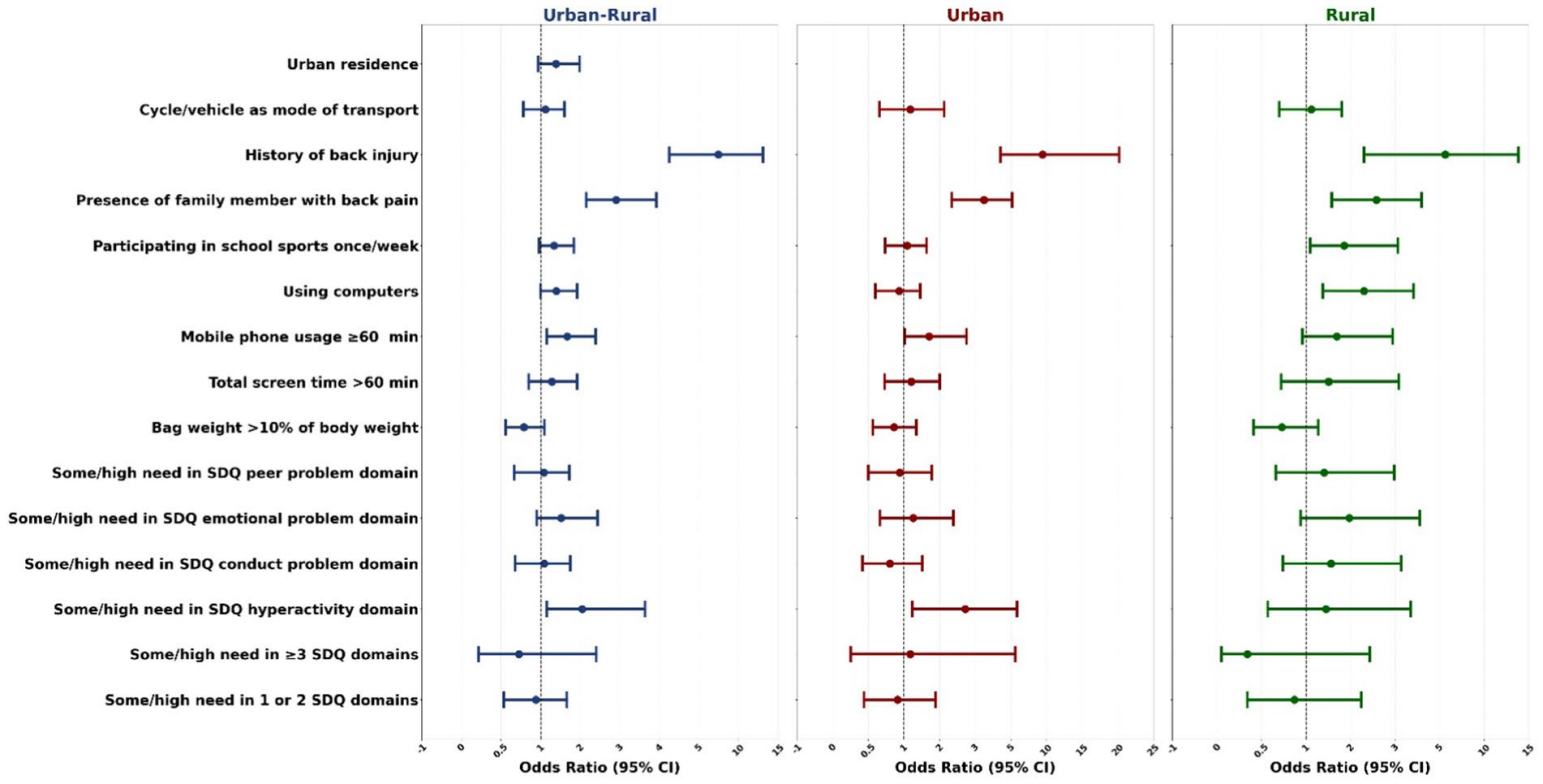
Multivariate regression model showing factors associated with nonspecific back pain according to residence.

A multivariate regression model was created using the significant risk factors of back pain from bivariate analysis, namely urban residence, history of back injury, presence of a family member with back pain, once-a-week participation in school sports, bag weight >10% of the student’s body weight, cycling or being driven to school, using computers, mobile phone usage ≥60 min, total screen exposure time >60 min, some/high need on SDQ emotional problem, SDQ conduct problem, SDQ hyperactivity, and SDQ peer problem domains and the total number of domains requiring some/high need. The selected method was Forward Wald. The same regression model was applied to urban and rural students separately to determine the differences in the risk factors (Figure 5).

As seen in table Figure 5, when the combined urban and rural data were considered, the significant risk factors of back pain were some/high need in the SDQ hyperactivity domain [OR 2.05, 95% CI 1.1 to 3.6], mobile use of ≥ 60 min [OR 1.67, 95% CI 1.1 to 2.4], using computers [OR 1.4, 95% CI 1.0 to 1.9], presence of a family member with back pain [OR 2.91, 95% CI 2.1 to 3.9] and history of back injury [OR 7.46, 95 CI 4.2 to 13.0]. For the urban adolescents, the significant factors were some/high need in the SDQ hyperactivity domain [OR 2.72, 95% CI 1.2 to 5.8], mobile usage ≥ 60 min [OR 1.71, 95% CI 1.0 to 2.7], presence of a family member with back pain [OR 3.49, 95% CI 2.3 to 5.2], and history of back injury [OR 9.43, 95% CI 4.4 to 20.1]. For the rural adolescents, the significant factors were using computers [OR 2.3, 95% CI 1.3 to 3.8], participation in school sports once a week [OR 1.85, 95% CI 1.1 to 3.1], presence of a family member with back pain [OR 2.58, 95% CI 1.6 to 4.2], and history of back injury [OR 5.65, 95% CI 2.3 to 13.9]. The results of the regression analysis have been tabulated in Supplementary Tables 1A–C.

## Discussion

The prevalence of nonspecific back pain in adolescents in our study (38.5%) is in agreement with many studies from India and abroad. The associated range of physical and psychological factors vary across urban and rural settings. In our study, the location of the pain was majorly in the upper back, of mild to moderate intensity, and lasted up to 2 days. This is in contrast to studies that have reported low back pain (16, 17). We did not find any association with female sex, higher ages, or BMI. The evidence remains divided regarding these factors (1, 11, 16–18). The bag weight continues to be an often-cited culprit for back pain in several studies, especially from India (13, 14, 19).

## Associated physical and psychological factors

Back pain in a child is a multifactorial phenomenon, not associated solely with the weight of the school bag. Although school bag weight of >10% of the body weight was found to be significantly associated with back pain in our study, it ceased to remain so when included in the multivariate regression model. This is consistent with pragmatic scenarios where an outcome is rarely the result of factors acting in isolation, but usually a manifestation of a myriad of factors interacting together and contributing collectively. Similar to previous studies, we have also found an association between a history of back injury and back pain (20). It has been previously reported that children report back pain when a family member complains of back pain (21, 22). Although the exact reason is unknown, it is probably because a child emulates adult behavior and imbibes adult traits.

Psychological factors are known to be associated with back pain in children (11, 23–25) with back pain being a physical manifestation of psychological or emotional problems (26). According to Engel’s biopsychosocial model, back pain is an interplay of biological, psychological, and social factors (27, 28). Our study reinforces this finding. While treating adult back pain, in addition to physical treatment modalities, addressing associated psychological factors is found to be effective (29). Similarly, the possibility of co-existence of psychological factors needs to be considered while evaluating a child with back pain.

In our study, screen time, especially mobile phone usage for ≥60 min a day, was found to be a significant risk factor of back pain. A recent meta-analysis has found a linear relationship between screen time exposure and back pain (30). Faulty posture while using the visual display units could be a possible reason, though not explored in our study.

## Adolescents’ perceived causes of back pain

A significantly greater proportion of urban children perceived that their back pain was due to their “heavy” school bag, reflecting the strong societal perceptions and information in the lay press. In contrast, a greater proportion of rural children believed that heavy domestic and farm work was the cause of their back pain. Rural childhood is different from growing up in a city in India. Among others, the important differences are in the school and domestic environments, family structures, and resources available to the child, such as public infrastructure and facilities. For various familial and social reasons, rural children in India continue to be engaged in domestic work involving washing utensils and clothes, fetching water from considerable distances, carrying heavy loads, and toiling on their farms (31). Also, rural children in our study have documented walking long distances to reach their school and bad road conditions as reasons for back pain. We agree with the other studies that the majority of back pain is nonspecific (6, 18, 32). This was confirmed by clinically examining the children in our study. Evidence generated from this study points to the need for a comprehensive intervention tailored to address the multitude of factors associated with back pain in adolescents. The substantial prevalence justifies the attention and engagement of all the stakeholders, from policymakers and public health researchers to educationists, clinicians, parents, and children in tackling this problem.

## Limitations and strengths

Our study has some limitations, including the inherent shortcomings of a cross-sectional design. Probability sampling was not employed for school selection. Schools granting permission were included. Recall bias could have crept in while reporting a week’s screen time exposure, and the details of back pain over the past month. We did not address posture, sleep patterns, sports activities outside school hours, ergonomics, and depression. Given the resources at our disposal, we had to restrict our study to one Indian state, which is not representative of the entire country. The Marathi version of SDQ needs reliability and validity testing.

Our study adds several new dimensions to the existing understanding of back pain in children in this age group. India has a huge rural population. However, back pain in rural Indian children remains a neglected topic. This is the first study to examine back pain in both, rural and urban adolescents taking into consideration physical and psychological factors strengthened with an on-site clinical examination. Also, our study has highlighted adolescents’ perceived reasons for their back pain.

## Conclusion

The prevalence of nonspecific back pain in urban and rural school-going adolescents is considerable and warrants public health attention. While assessing an adolescent with nonspecific back pain, the focus needs to be broadened to consider psychological factors and screen time exposure, and not only the school bag weight. Appropriate interventions at the individual and school levels can be devised to address these modifiable factors associated with nonspecific back pain in this population.

## Data Availability

The raw data supporting the conclusions of this article will be made available by the authors, without undue reservation.
