# Supplementary material for: Nonspecific back pain in adolescents, its associated physical and psychological factors and urban–rural differences: a cross-sectional analytical study
Source: Front Public Health. 2026 Mar 13;14:1780994. doi: 10.3389/fpubh.2026.1780994 (PMC13021778; doi:10.3389/fpubh.2026.1780994)
Supplement: Supplementary file 1 [file Table_1.DOCX]

**Supplementary Table 1A:** Multivariate regression analysis for urban-rural combined data

|  | **OR** | **95% CI** | | **p-value** |
| --- | --- | --- | --- | --- |
|  |  | **Lower** | **Upper** |  |
| Some/high need in 1 or 2 SDQ domains | 0.94 | 0.54 | 1.66 | 0.850 |
| Some/high need in ≥3 SDQ domains | 0.73 | 0.22 | 2.41 | 0.614 |
| Some/high need in SDQ hyperactivity domain | 2.05 | 1.15 | 3.64 | 0.014 |
| Some/high need in SDQ conduct problem domain | 1.09 | 0.68 | 1.75 | 0.698 |
| Some/high need in SDQ emotional problem domain | 1.52 | 0.95 | 2.44 | 0.076 |
| Some/high need in SDQ peer problem domain | 1.08 | 0.67 | 1.73 | 0.751 |
| Bag weight >10% of body weight | 0.79 | 0.56 | 1.10 | 0.173 |
| Total screen time >60 min | 1.28 | 0.85 | 1.92 | 0.227 |
| Mobile phone usage ≥60 min | 1.67 | 1.15 | 2.39 | 0.007 |
| Using computers | 1.40 | 1.00 | 1.92 | 0.048 |
| Participating in school sports once/week | 1.34 | 0.98 | 1.84 | 0.059 |
| Presence of family member with back pain | 2.91 | 2.16 | 3.92 | 0.000 |
| History of back injury | 7.46 | 4.25 | 13.09 | 0.000 |
| Cycle/vehicle as mode of transport | 1.12 | 0.78 | 1.61 | 0.508 |
| Urban residence | 1.39 | 0.97 | 1.99 | 0.070 |
| Constant | 0.10 |  |  | 0.000 |

**Supplementary Table 1B:** Multivariate regression analysis for urban data

|  | **OR** | **95% CI** | | **p-value** |
| --- | --- | --- | --- | --- |
|  |  | **Lower** | **Upper** |  |
| Some/high need in 1 or 2 SDQ domains | 0.91 | 0.44 | 1.888 | 0.805 |
| Some/high need in ≥3 SDQ domains | 1.18 | 0.25 | 5.631 | 0.830 |
| Some/high need in SDQ hyperactivity domain | 2.72 | 1.23 | 5.846 | 0.013 |
| Some/high need in SDQ conduct problem domain | 0.80 | 0.42 | 1.517 | 0.505 |
| Some/high need in SDQ emotional problem domain | 1.26 | 0.66 | 2.394 | 0.469 |
| Some/high need in SDQ peer problem domain | 0.94 | 0.50 | 1.778 | 0.864 |
| Bag weight >10% of body weight | 0.86 | 0.561 | 1.348 | 0.531 |
| Total screen time >60 min | 1.20 | 0.728 | 2.004 | 0.464 |
| Mobile phone usage ≥60 min | 1.71 | 1.013 | 2.757 | 0.044 |
| Using computers | 0.93 | 0.597 | 1.456 | 0.757 |
| Participating in school sports once/week | 1.09 | 0.732 | 1.630 | 0.665 |
| Presence of family member with back pain | 3.49 | 2.346 | 5.186 | 0.000 |
| History of back injury | 9.43 | 4.415 | 20.172 | 0.000 |
| Cycle/vehicle as mode of transport | 1.18 | 0.657 | 2.131 | 0.575 |
| Constant | 0.19 |  |  | 0.000 |

**Supplementary Table 1C**: Multivariate regression analysis for rural data

|  | **OR** | **95% CI** | | **p-value** |
| --- | --- | --- | --- | --- |
|  |  | **Lower** | **Upper** |  |
| Some/high need in 1 or 2 SDQ domains | 0.87 | 0.34 | 2.24 | 0.784 |
| Some/high need in ≥3 SDQ domains | 0.34 | 0.05 | 2.43 | 0.287 |
| Some/high need in SDQ hyperactivity domain | 1.45 | 0.57 | 3.70 | 0.432 |
| Some/high need in SDQ conduct problem domain | 1.56 | 0.74 | 3.28 | 0.239 |
| Some/high need in SDQ emotional problem domain | 1.97 | 0.94 | 4.12 | 0.072 |
| Some/high need in SDQ peer problem domain | 1.41 | 0.66 | 2.98 | 0.367 |
| Bag weight >10% of body weight | 0.73 | 0.41 | 1.28 | 0.276 |
| Total screen time >60 min | 1.51 | 0.72 | 3.17 | 0.270 |
| Mobile phone usage ≥ 60 min | 1.69 | 0.96 | 2.95 | 0.065 |
| Using computers | 2.30 | 1.38 | 3.83 | 0.001 |
| Participating in school sports once/week | 1.85 | 1.10 | 3.12 | 0.019 |
| Presence of family member with back pain | 2.58 | 1.58 | 4.19 | 0.000 |
| History of back injury | 5.65 | 2.30 | 13.88 | 0.000 |
| Cycle/vehicle as mode of transport | 1.12 | 0.70 | 1.80 | 0.618 |
| Constant | 0.059 |  |  | 0.000 |

SDQ: Strength and Difficulties Questionnaire
